# Regulatory Variants and Disease: The E-Cadherin −160C/A SNP as an Example

**DOI:** 10.1155/2014/967565

**Published:** 2014-09-02

**Authors:** Gongcheng Li, Tiejun Pan, Dan Guo, Long-Cheng Li

**Affiliations:** ^1^Department of Urology, Wuhan General Hospital, Guangzhou Command PLA, China; ^2^Molecular Medicine Laboratory, Peking Union Medical College Hospital, Chinese Academy of Medical Sciences and Peking Union Medical College, Beijing, China; ^3^Department of Urology, University of California San Francisco, San Francisco, CA 94158, USA

## Abstract

Single nucleotide polymorphisms (SNPs) occurring in noncoding sequences have largely been ignored in genome-wide association studies (GWAS). Yet, amounting evidence suggests that many noncoding SNPs especially those that are in the vicinity of protein coding genes play important roles in shaping chromatin structure and regulate gene expression and, as such, are implicated in a wide variety of diseases. One of such regulatory SNPs (rSNPs) is the E-cadherin (CDH1) promoter −160C/A SNP (rs16260) which is known to affect E-cadherin promoter transcription by displacing transcription factor binding and has been extensively scrutinized for its association with several diseases especially malignancies. Findings from studying this SNP highlight important clinical relevance of rSNPs and justify their inclusion in future GWAS to identify novel disease causing SNPs.

## 1. Introduction

Genetic variation contributes to virtually every human disease, conferring susceptibility or resistance or influencing interaction with environmental factors [1]. The most common type of human genetic variation is single nucleotide polymorphism (SNP), where two alternative bases occur at appreciable frequency (>1%) in the human population [2]. As of NCBI dbSNP Build 141 (http://www.ncbi.nlm.nih.gov/SNP/), there are about 43 million validated SNPs in human genome occurring about once in every 72 basepairs (bp). While much focus has been given to SNPs in coding sequences in genome-wide association studies (GWAS), the role of noncoding SNPs, which count more than coding SNPs, is much less studied. Many such noncoding SNPs that reside in the noncoding sequences (e.g., promoters, enhancers, and 3′ termini) surrounding protein coding genes have been shown to have profound effects on the expression of neighboring genes and can cause disease phenotypes [3, 4] and are thus called regulatory SNPs (rSNPs) [5, 6].

In 2000, when we were mapping DNA methylation in the CpG island region of the E-cadherin promoter in cancer samples using the bisulfite genomic sequencing technique [7], we accidently identified a novel C/A polymorphic site at the −160 location of the E-cadherin promoter within the mapped region. Further molecular characterization revealed that the two alleles confer the E-cadherin promoter different transcriptional activities. Since then, this SNP (reference SNP accession rs16260) has been extensively scrutinized for its association with different types of cancer and several noncancerous diseases (Table 1) by worldwide groups including our own [8, 9]. In this review, we summarize data accumulated in the past 13 years on the association of the E-cadherin −160C/A SNP with human conditions and highlight the important function of rSNPs as a risk factor for diseases. Nevertheless, this review is not intended to serve as a meta-analysis, many of which have already been published [10–13].

## 2. Regulatory Variants and Gene Expression

Unlike coding SNPs that either cause a change in amino acid sequences or do nothing, rSNPs may have an effect on the level of transcription of neighboring genes. Multiple mechanisms can be attributed to such effect including affecting binding affinity of protein transcription factor or altering promoter methylation [14]. It is also likely that rSNPs affect sequence specific binding of nonprotein transcriptional factor such as noncoding RNA. In this regard, it has recently been shown that miRNAs and long noncoding RNAs (ncRNAs) can regulate gene transcription or chromatin structure in a sequence-dependent fashion [15]. Some rSNPs have such a profound effect on gene transcription so as to create a new transcriptional promoter which directly contributes to the etiology of α-thalassemia, a genetic disease [16].

Normal variation in gene expression is common among individuals and can be attributed to genetic factors [17]. However, the underlying molecular mechanisms have remained unclear until recently when several genome-wide studies highlight the importance of regulatory variants in affecting gene expression by altering transcription factor binding and chromatin structure [18–21]. Epigenetic code has been known to underlie critical biological processes ranging from development, differentiation, and disease. However the fundamental question that remains unanswered is how epigenetic code* per se* is established and regulated [22]. After all, genetics still underlie epigenetic mechanisms of gene regulation. By combinatorial analysis of gene expression data and binding profiles of NF*κ*B and RNA polymerase II (RNAP II), Kasowski et al. found extensive contribution of genetic variation to variation in TF binding, many of which can affect gene expression and are thus functional [18]. Similarly, McDaniell et al. found that individual-specific and allele-specific variation in chromatin structure and transcription factor binding can be transmitted from parents to children as a result of genetic variation [19]. Very recently, Kasowski et al. and Kilpinen et al. further showed that the mechanism underlying chromatin variation resulting from genetic variability is mainly through disrupting TF binding [20, 21].

## 3. The Function of E-Cadherin Gene

Epithelia are essential and abundant tissues in most eukaryotic organs, and over 90% of the malignant human tumors are derived from epithelia [23]. Development of malignant tumors is in part characterized by the ability of tumor cells to overcome cell-cell adhesion and to invade surrounding tissues [24]. E-cadherin, one of the classic cadherins, playing a major role in the establishment and maintenance of intercellular adhesion, cell polarity, and tissue architecture [25], has been implicated in carcinogenesis because it is frequently lost or downregulated in human epithelial cancers including prostate, breast, bladder, pancreas, stomach, and colon tumors [26–30]. Compelling evidence also indicates that E-cadherin is a potent tumor invasion suppressor [24, 31] by inhibiting epithelial to mesenchymal transition (EMT) [32].

The molecular mechanisms underlying the loss of E-cadherin expression in carcinomas are not fully understood. Somatic mutations in the E-cadherin gene have been identified in diffuse gastric carcinomas [33] and lobular breast carcinomas [34] and in a small proportion of gynecologic cancers [35]. However, in the majority of cancers, where E-cadherin expression is downregulated, the molecular mechanisms underlying this defect are still poorly understood. A major mechanism leading to the decrease in E-cadherin expression seems to result from a decrease in transcription [24, 36, 37], since mutations within the E-cadherin coding sequence have been reported as rare in breast, gastric, and gynecological cancers [34]. Additionally, inactivation of E-cadherin has been associated with hypermethylation of CpG islands within the proximal promoter region of the E-cadherin gene in a number of human cancers [7, 38, 39].

Dysfunction of E-cadherin has also been associated with a number of nonmalignant diseases such as ulcerative and Crohn's colitis, Langerhans' cell histiocytosis, endometriosis, and autosomal dominant polycystic kidney disease [40, 41].

## 4. E-Cadherin −160C/A SNP Affects E-Cadherin Transcriptional Activity

**Figure 1:**
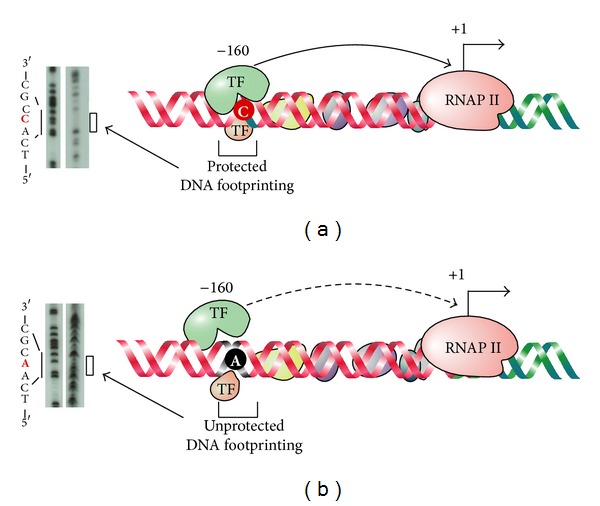
rSNPs regulate gene transcription by affecting transcription factor (TF) binding. rSNPs in regulatory sequences such as gene promoters may affect gene expression at the transcriptional level and this regulation is mainly realized through affecting transcription factor binding. In the example of −160C/A SNP in E-cadherin promoter, the −160 location is the binding site of putative TFs. The C allele of this site allows for binding of the TFs, as evidenced by a protected footprint on DNA footprinting assay, leading to active transcription of E-cadherin gene (a), whereas the A allele prevents the TFs from binding likely due to steric hindrance, resulting in the loss of footprint of the TFs and attenuated transcription (b) [42].

The E-cadherin −160C/A SNP is located at the −160 location relative to the transcription start site (TSS) of E-cadherin. Cloning the two alleles into the upstream of a promoterless luciferase reporter gene revealed that the A allele decreases transcriptional activity by 68% compared with the C allele in a reporter gene analysis, suggesting that the A allele may reduce E-cadherin expression* in vivo* [42]. This finding is supported by other studies that reported similar reduced transcriptional activity from the A allele [43, 44]. Based on footprinting and gel shift assays, the −160 site is probably bound by two protein complexes and the two alleles have very different binding affinity for nuclear proteins with the C allele bound by more proteins than the A allele as revealed by gel shift assay. Footprinting assay confirmed that only the C allele is protected from DNase digestion at the polymorphic site. The protected region contains a 7-nucleotide sequence which may be the binding site for unknown transcription factors that are required for achieving higher transcriptional activity (Figure 1).

By bioinformatics analyses using the TFSEARCH and TESS databases, Borges Bdo et al. identified putative binding sites at the −160 location for RAR-*β*, ER-α, AP-1, StuAp, and CF-1. When the −160 C is changed to A, the binding site for CF-1 is eliminated and a putative de novo binding site is created for two transcription factors: RC2 and MCBF [45].

The decreased transcriptional activity from the A allele may be explained as the result of structure differences between the A and the C alleles, which hinders the access of DNA by transcription factors. However, the change of a cytosine to an adenosine in the DNA structure does not abandon the binding completely (Figure 1).

By analyzing E-cadherin protein expression in tissue samples, Kuraoka et al. showed that samples with C/C genotype have higher E-cadherin protein expression than C/A genotype [46], despite the fact that CC genotype is associated with higher risk of gastric cancer [46]. Similarly, expression of E-cadherin protein as assessed by immunohistochemistry and western blotting is lower in endometrium tissues of endometriosis patients carrying the A allele [47]. There is, so far, no enough evidence to indicate that the two alleles have an impact on E-cadherin expression* in vivo*. Further studies are needed to verify whether this SNP has an impact on E-cadherin expression* in vivo*.

## 5. E-Cadherin rSNP and DNA Methylation

It has been reported that SNPs can alter CpG methylation [48–50], representing one of the mechanisms that link genetic alternations to epigenetic changes. This view is corroborated by a recent genome-wide DNA methylation mapping study in which differentially methylated regions (DMRs) are found to contain enriched SNPs associated with cell-type related diseases revealed by GWAS [14]. Although the exact mechanism is unknown, differential protein/transcription factor binding can presumably contribute to the differential methylation profiles between different alleles, especially when a SNP occurs within a CpG site. In this regard, Borges Bdo et al. correlated −160C/A alleles with DNA methylation status in Brazilian gastric cancer patients and found that the −160A allele is positively associated with hypermethylation at the E-cadherin promoter and also with increased risk of developing gastric cancer [45]. However, in another study of Japanese gastric patients, the C/C genotype was found to be associated with higher risk of gastric cancer and higher E-cadherin expression but not associated with E-cadherin promoter hypermethylation [46]. This discrepancy might have arisen from disease stages/grades and the ages of the patients since those variables are known to be determinants of promoter hypermethylation [51].

## 6. E-Cadherin −160C/A SNP Genotype Frequency in General Populations

Based on data from the 1000 genome phase I population consisting of 1094 worldwide individuals, the global minor allele frequency (MAF) for A in the −160C/A SNP is 0.2323. The frequency varies considerably among ethnic groups with the lowest A allele frequency of 5.3% found in an Asian population (Coriell Cell Repositories, 38 chromosome counts) and the second lowest of 10.2% found in a population of African ancestry in southwest USA (98 chromosome counts). The highest A allele frequencies of 32.6% and 31.0% are found, respectively, in the Hispanics (46 chromosome counts) and Centre d'Etude du Polymorphisme Humain (CEPH) pedigrees [UTAH (93%), French (4%), and Venezuelan (3%)] (chromosome counts 184).

## 7. E-Cadherin −160C/A SNP and Cancer

The association of −160C/A SNP with various types of cancer has been extensively studied. As of April, 2014, there are at least 49 case-control studies examining the association of this SNP with gastric, prostate, bladder, breast, colorectal, nasopharyngeal, endometrial, pancreatic, cervical, lung, oral, liver, thyroid, and ovarian cancer and lymphoma (Table 1). At least 15 meta-analysis studies have been published with the most recent one summarizing 47 cancer-related case-control studies [10]. Results from these studies reveal that −160 SNP is a cancer type specific and also ethnicity specific risk factor.

### 7.1. E-Cadherin −160C/A SNP and Urological Cancer of the Prostate and the Bladder

The first-ever study associating −160C/A SNP with cancer risk was published in 2002 [52]. The authors genotyped 82 patients with localized prostate cancer including 57 with sporadic prostate cancer and 25 with hereditary prostate cancer and 188 controls from a Dutch population and found that carriers of the A had a 3.6-fold increased risk for prostate cancer compared to C-only carriers. Interestingly, heterozygous (CA) genotypes had an almost 4-fold increased risk of prostate cancer compared to CC genotype whereas homozygous (AA) had only a 1.7-fold increased risk. In addition, the A allele and AA/CA genotypes render less risk for hereditary prostate cancer than for sporadic prostate cancer. This first study was then followed by 9 others examining a total of 3,570 cases and 3,304 controls as summarized in the meta-analysis by Wang et al. [10]. These studies have found that the A allele is associated with higher risk for prostate cancer in the Europeans (OR = 1.56; 95% CI = 1.16–2.08) and Asians (OR = 1.10; 95% CI = 0.86–1.41), but not in black and white Americans [10].

Three case-control studies have observed that the A allele of E-cadherin C/A SNP confers higher risk for bladder cancer in the Chinese [53], Japanese [54], and Dutch [55] and is associated with invasive cancer [53]. Of particular note is a clinical outcome study following 302 patients with superficial bladder cancer after transurethral resection of the tumors for a median follow-up of 27.65 months [56]. Among 274 Caucasians in the cohort, 50% developed recurrence during the follow-up period. Compared to patients with CC genotype, patients carrying at least one A allele had a 32% reduction in recurrence risk (adjusted HR 0.68; 95% CI 0.48–0.96).

### 7.2. E-Cadherin −160C/A SNP and Gastrointestinal Tract Cancer

E-cadherin −160C/A SNP has been studied most intensively in gastric cancer resulting in at least 15 case-control and 6 meta-analysis studies. Findings from these studies suggest that −160C/A SNP is an ethnical dependent risk factor for gastric cancer. Interestingly, in Asian population, this SNP may be reversely associated with gastric cancer risk with the A allele possessing a protective effect on developing gastric cancer [46]. However, a recent study directly sequencing 167 gastric cancer (107 diffuse and 60 intestinal) cases and 134 controls in a Chinese population found that the −160 A allele was significantly higher in diffuse gastric cancer cases (OR 1.75, 95% CI, 1.014–3.022) [57].

### 7.3. E-Cadherin **−** 160C/A SNP and Cancer Metastasis

In a Brazilian study, the AA genotype is associated with a higher risk of metastatic disease at diagnosis (OR 3.43; 95% CI 1.27–9.27; *P* = 0.023) [58]. In a Japanese population of 106 gastric cancer cases, which had a higher CC genotype frequency compared to controls, patients positive for lymph node metastasis had a further higher CC genotype frequency than those without metastasis (OR 2.86; 95% CI 1.28–6.36; *P* = 0.01) [46]. The CC genotype in cases is significantly associated with poorly differentiated adenocarcinoma, deep invasion, and lymph node metastasis [46]. However, other studies could not identify an association of −160C/A SNP with lymphatic metastasis in esophageal squamous cell carcinoma, gastric cardia adenocarcinoma, [59] and nasopharyngeal cancer [60].

## 8. E-Cadherin −160C/A SNP and Noncancerous Diseases

While most studies on the −160C/A SNP focused on cancer, a few have examined its association with noncancerous diseases including orofacial clefts, asthma, urolithiasis, endometriosis, and infection. Song et al. genotyped 140 nonsyndromic orofacial clefts (NSOC) cases and 107 healthy individuals from a Chinese Han population. Although there is lack of association between this SNP and overall risk of NSOC, when all cleft cases were stratified into four groups (i.e., cleft lip with or without cleft palate, cleft lip only, cleft lip with cleft palate, and cleft palate only), the −160C/A SNP overall genotype frequencies in cleft palate only (CPO) groups were significantly different from those in the controls (*P* = 0.004) and AA genotype significantly increased the risk of CPO by 5.90-fold (OR 6.90; 95% CI 1.47–32.40), suggesting that E-cadherin activity may contribute to etiology of CPO.

Govatati et al. [47] studied the association of −160 SNP with endometriosis in Indian women (715 cases and 500 controls) and found that the −160A/A frequencies are higher in cases than in control (*P* < 0.0019). In another case-control study performed in Japanese women (520 cases and 520 healthy controls), no such association, however, was found [61].

It is known that levels of E-cadherin can affect airway remodeling which is a feature of chronic asthma and is characterized by an increased turnover of cells and extracellular matrix [62]. Very recently, Wang et al. studied the effects of environmental tobacco smoke (ETS) and E-cadherin −160C/A SNP on the risk of developing childhood asthma in 299 asthmatic children and 383 healthy controls. They found that EST exposure to more than 5 cigarettes/day and the presence of CDH1 AA/CA genotypes had a significantly increased risk for childhood asthma (OR 1.53; 95% CI 1.08–2.17), suggesting a role of gene and environment interactions in asthma risk [63].

In a hospital-based case-control study of 127 nephrolithiasis patients and 152 controls, Tan et al. genotyped the −160C/A SNP and found that CA/AA genotypes are associated with a significantly decreased risk of nephrolithiasis (OR = 0.53; 95% CI = 0.32–0.87), compared with the CC genotype. The association is even greater among subgroups of BMI > 24 kg/m^2^ (OR = 0.38; 95% CI = 0.17–0.85), age ≤ 57 years (OR = 0.47; 95% CI = 0.24–0.93), and men (OR = 0.56; 95% CI = 0.29–0.99) [64].

Genetic variation is known to affect susceptibility to infection. In an effort to examine genetic risk factors for postinfectious irritable bowel syndrome (PI-IBS), Villani et al. genotyped 71 functional variants including −160C/A SNP which, among the other 2, is an independent risk factor for developing PI-IBS [65]. Since E-cadherin is a transmembrane glycoprotein which forms the tight junctions with apical junctional complex which provides intestinal barrier function, decreased E-cadherin expression may contribute to PI-IBS symptoms by increasing intestinal permeability.

## 9. Concluding Remarks

Results from recent genome-wide sequencing analysis highlight the importance of rSNPs in modulating neighboring gene expression by affecting transcription factor binding and chromatin structure [20, 21]. Intensive studies in the past decade on the E-cadherin −160C/A rSNP have revealed that this rSNP can modify the risk of a number of diseases, especially gastric, prostate, and bladder cancer. In certain tumor types and ethnical groups, however, there are inconsistent results regarding the effect of the A allele on disease risk. It is possible that other nearby rSNPs in haplotype with −160C/A could mask the effect of the latter. In this regard, additional SNPs in the E-cadherin promoter have been reported such as the −347G/GA which could also modify promoter transcriptional activity and disease risk [66–68]. Future GWAS studies that include the −160 rSNPs as well as others in E-cadherin promoter are needed to further clarify the functional role of E-cadherin −160C/A SNP in diseases.

## Figures and Tables

**Table 1:** Association of E-cadherin −160C/A SNP and diseases.

Disease	Case/control	Ethnicity	Positive association	No association
Gastric cancer	505/246	Italian	[44]	
	239/343	Chinese	[59]	
	387/392	Chinese	[69]	
	107/134	Chinese	[57]	
	201/196	Chinese	[70]	
	245/950	European	[71]	
	39/78	Mexican	[72]	
	153/303	Japanese	[73]	
	106/90	Japanese	[46]	
	53/70	Italian	[74]	
	192/170	Omani	[75]	
	412/408	Italian		[76]
	572/589	Chinese		[68]
	206/261	Chinese		[77]
	292/146	Korean		[78]
	433/466	Canadian, German, Portuguese		[79]

Prostate cancer	82/188	Dutch	[52]	
	1036/669	Swedish	[80]	
	183/168	Slovenian	[81]	
	200/159	Japanese	[82]	
	236/209	Japanese	[83]	
	801/1636	Swedish	[84]	
	86/126	Caucasian	[9]	
	49/117	African American		
	219/102	European American	[85]	
	119/112	African American		[85]
	89/123	Jamaicans		
	98/0	Caucasian		[86]
	219/219	Japanese		[54]

Bladder/urothelial cancer	50/50	Chinese	[8]	
	180/100	Chinese	[53]	
	314/314	Japanese	[54]	
	197/344	Dutch	[55]	
	302/0	Caucasian, African American, Hispanic	[56]	

Colorectal cancer	194/220	German	[87]	
	5679/5412	British	[88]	
	130/130	Brazilian	[58]	
	505/246	Italian	[44]	
	334/171	British		[89]

Pancreatic cancer		Chinese	[90]	

Nasopharyngeal cancer	302/140	Tunisian	[60]	

Ovarian cancer	207/256	Chinese	[91]	

Renal cancer	526/514	Polish		[92]

Lung cancer	95/85	Chinese	[93]	

Hepatocellular carcinoma	131/347	Chinese	[94]	
	93/0	Chinese		[95]

Benign diseases				

Endometriosis	505/246	Italian	[44]	
	715/370	Indian	[47]	
	511/498	Japanese		[61]
	152/189	Chinese		[96]

Nonsyndromic orofacial clefts (NSOC)	140/107	Chinese	[97]	

Asthma	299/383	Asian	[63]	

Nephrolithiasis	127/152	Chinese	[64]	

PI-IBS	228/581	Caucasians	[65]	
